# Supplementary material for: IL-18/IL-37/IP-10 signalling complex as a potential biomarker for discriminating active and latent TB
Source: PLoS One. 2019 Dec 10;14(12):e0225556. doi: 10.1371/journal.pone.0225556 (PMC6903724; doi:10.1371/journal.pone.0225556)
Supplement: Attachment — Submitted filename: Response to reviewers.docx [file pone.0225556.s004.docx]

To Reviewer #1:

We would like to thank you kindly for reading our manuscript “IL-18/IL-37/IP-10 signaling complex as a potential biomarker for discriminating active and latent TB” and for all the questions and suggestions.

Our answers to the questions are as follows:

*1. Line 130 Fifty-two % should be replaced by fifty-five% according to the number in brackets.*

We modified the sentence as suggested (p.6, line 130).

*2. Line 187 I assume that "Me" is the abbreviation for median but it was not introduced previously in the manuscript.*

The abbreviation was introduced in the sentence where it first appeared (p.8, line 187)

*3. Line 210 I suppose that average should be replaced by median.*

The word “average” was removed (p. 9, line 210)

*4. Line 285 please write also the CI of AUC for HC vs ATB.*

It was added (p. 12, line 285)

*5. Line 365 and fig 5 the group ATB in the manuscript is written TB in the figure 5, could you please replace TB with ATB in the figure.*

It was corrected.

*6. Line 414: in the manuscript the authors mentioned “squares” as symbol for IGRA negative patients in figure 6, but I don’t see squares in figure 6, only circles of different colours. The authors should fix this point.*

It was fixed (p. 18, lines 415-418)

*7. Lines 488, 489, 490 and 493 please write CD4+ with the symbol + superscript.*

The “+” symbol was written with the superscript (p.21, lines 490, 491, 492, 495).

To Reviewer #3:

We would like to thank you kindly for reading our manuscript “IL-18/IL-37/IP-10 signaling complex as a potential biomarker for discriminating active and latent TB” and for all the questions and suggestions.

Our answers to the questions are as follows:

*1. - P-values in the text (lines 209 to 215, p-values are still shown with two -rather than three- decimal places)*

We shown p-values in the text with three decimal places (p.9, lines 209-215).

*2. - CI values in table 4 (IFN-gamma/HC vs LTBI CI)*

It was added.

*3. - Table 6 (decimals and n)*

It was corrected.

*4. Tables (e.g. tables 3 and 5) should show always the sample sizes involved in comparisons and correlations, regardless this information has already given in the text*

The information on the sample sizes was added.

*5. Table 7: please show the SE of the coefficients or their CI*

The CI’s of the coefficients were added.
